# Innovative Biobased Thermoplastic Binders for Sustainable Lithium-Ion Batteries

**DOI:** 10.1021/acsomega.5c00341

**Published:** 2025-06-02

**Authors:** Daniela de Morais Zanata, Rafael Del Olmo, Mikel Larumbe, Marcela de Paula Ramos, Nery M Aguilar, Irune Villaluenga

**Affiliations:** † POLYMAT, Applied Chemistry Department, Faculty of Chemistry, University of the Basque Country UPV/EHU, 20018 Donostia-San Sebastián, Spain; ‡ IKERBASQUE Basque Foundation for Science, 48013 Bilbao, Spain

## Abstract

The growing global demand for batteries has driven the search for sustainable materials for energy storage applications. One promising approach to enhance the sustainability of lithium-ion batteries (LIBs) is replacing poly­(vinylidene fluoride) (PVDF) with biobased polymers as binders. However, the primary challenge lies in identifying biobased binders that can meet the mechanical and electrochemical performance requirements necessary for high-efficiency batteries. Balancing sustainability with performance remains an obstacle in the development of biobased alternatives. This study explores the potential of novel biobased polymers to replace PVDF as a binder in LFP cathodes. Biobased polymers, synthesized from isosorbide and poly­(ethylene glycol) (PEG), were developed, and the results show that cathodes using the ISB-25PEG binder exhibit superior adhesion, cohesion, and battery performance that is comparable to that of the traditional PVDF binder. These findings highlight the potential of ISB-25PEG as a sustainable and high-performance alternative to the next generation of energy storage devices, marking an important step forward in the development of environmentally friendly battery technologies.

## Graphical abstract

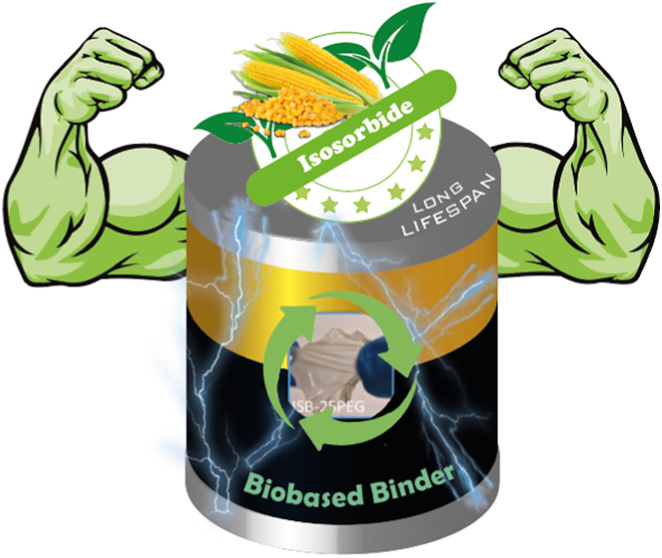

## 1 Introduction

The rapid growth in global energy demand encourages the transition from fossil fuels to renewable energy storage technologies.^1^ Lithium-ion batteries (LIBs) are crucial in this transition, owing to their high energy density, long cycle life, and versatility across various applications.^2,3^ Despite their broad advantages, the sustainability in LIB technologies remains a significant challenge.^4,5^

A critical component of LIBs is the binder, which keeps active material particles and conductive agents fixed on the current collector, ensuring the structural integrity by the accommodation of the mechanical stresses and volumetric changes that occur during battery charge and discharge cycles.^6,7^ This is one of the reasons why poly­(vinylidene fluoride) (PVDF) has dominated the binder market in the last few years.^8,9^ However, the use of fluorine-based polymers has raised concerns, especially due to their negative environmental and health impact.^10^ For such reasons, the European Chemicals Agency (ECHA) proposed the restriction of the large-scale application of per- and polyfluoroalkyl substances (PFAS) in the future.^11^ Therefore, the substitution of PVDF with sustainable binders for LIBs has attracted widespread attention,^12–14^ although finding an environmentally friendly alternative that is low-cost and has excellent thermal and mechanical properties is still a challenge.

In recent years, increasing attention has been directed toward biobased binders, such as polysaccharides,^15,16^ lignin-based materials,^17^ or cellulose derivatives,^14,18^ as substitutes for synthetic ones. Carboxymethyl cellulose (CMC) is the most famous biobased binder used in the industry.^14^ Usually, CMC is combined with a rubberizing agent, such as styrene–butadiene rubber, to avoid the cracking and peeling of the electrode layer upon drying, especially if high loadings are used.^18^ Other biobased polymers, such as guar gum, potato starch, and wheat starch, showed high flexibility at high loadings, but they delaminated upon calendaring due to the low inner electrode cohesion.^16^ In summary, the key obstacle for biobased binders is to meet the mechanical and electrochemical performance standards required for high-efficiency LIBs.

This study addresses these challenges by investigating the potential of a novel biobased binder for cathodes in LIBs, composed of isosorbide (ISB) and poly­(ethylene glycol) (PEG). Isosorbide, a renewable diol derived from glucose,^19^ has been used as a building block in different polymers^20–23^ and offers excellent thermal stability and mechanical strength, while PEG (which can also be biobased^24^) contributes to flexibility and processability.^25^ The copolymerization of rigid ISB and PEG within a (co)­poly­(arylene ether sulfone) linear chain resulted in a thermoplastic biobased binder with a tunable *T*_g_, and therefore, low processing temperatures. It is important to note that these copolymers are not entirely biobased, as the arylene ether sulfone moiety is derived from petroleum. However, replacing bisphenol A (BPA)—commonly used in the large-scale production of polysulfones—with biobased diols represents a significant environmental advancement. This substitution reduces reliance on fossil fuel-derived materials while maintaining the performance of the polymers. Additionally, bis­(4-fluorophenyl) sulfone (DFPS), a key component for the production of the binders developed in this study, is not classified as a PFAS (per- and polyfluoroalkyl substance),^26^ unlike materials such as PVDF, ensuring compliance with current and future European regulations.

Additionally, a comparative analysis was conducted to benchmark the performance of ISB-PEG copolymer-based cathodes against PVDF-based ones. Key performance metrics, including binder adhesion, electrochemical stability, and overall battery efficiency, were assessed under conditions representative of practical battery operation. The cathodes based on ISB-25PEG not only match the performance of PVDF in terms of adhesion and stability but also enable the development of LIBs with improved sustainability. By elucidating the potential of ISB-PEG-based copolymers as a viable and sustainable alternative to PVDF, this study contributes to the development of environmentally friendly LIBs, contributing to future research and innovation in integrating renewable materials into next-generation energy storage devices.

## 2 Experimental Section

### 2.1 Materials

Isosorbide (ISB, 98%, TCI) and 18-crown-6 (99%, Thermo Scientific) were manipulated in a glovebox under an argon flow and used without further purification. Bis­(4-fluorophenyl) sulfone (DFPS, 99%, TCI), potassium carbonate (K_2_CO_3_, 99%, Acros Organics), anhydrous dimethyl sulfoxide (DMSO, 99.8%, Thermo Scientific), poly­(ethylene glycol) PEG (molecular weight of 1300 g mol^–1^, Sigma-Aldrich), and acetic acid (HPLC grade) were used without further purification. For comparative purposes, poly­(vinylidene fluoride) (PVDF Solef 5130, 1000–1100 kg mol^–1^) was employed as the reference binder with 1-methyl-2-pyrrilidone (NMP, Sigma-Aldrich 99%) as the solvent. For the processing of the isosorbide-based electrodes, dimethylformamide (DMF, Acros, 99.5%) was used. To prepare the electrode slurries, conducting carbon (Super C_65_, Timcal) and lithium iron phosphate (LiFePO_4_, Aleees) were used without further treatment. As the liquid electrolyte, a solution of 1 M LiPF_6_ in EC:DEC (1:1, vol %) (LP40, Solvionic, 99.9%) was used as received.

### 2.2 Synthesis of the SB Polymer and ISB-xPEG Copolymers

The SB polymer was synthesized following a similar procedure described by Park et al.^23^ For ISB-10PEG, ISB (0.5 g, 3.46 mmol), PEG (0.5 g, 0.39 mmol), DFPS (0.98 g, 3.85 mmol), and K_2_CO_3_ (0.66 g, 4.81 mmol) were dried at 40 °C overnight in a dried two-neck glass flask. A Dean–Stark apparatus was coupled to the system, which was previously purged with argon, before the addition of anhydrous DMSO (4 mL). Then, a solution of 18-crown-6 (0.051 g, 0.19 mmol) in anhydrous DMSO (1 mL) was added to a flask via a syringe under an argon atmosphere. The reaction mixture was heated at 155 °C for 24 h, under a mild argon flow. After polymerization, the reaction mixture was diluted with DMF (5 mL), cooled to room temperature, and precipitated into water containing acetic acid (2% v/v). The solid was filtered and reprecipitated in deionized water after dissolving in DMF. The polymer was dried under vacuum at 100 °C overnight. ISB-25PEG was prepared using the same procedure, adjusting the ISB (0.39 g, 2.70 mmol) and PEG (1.5 g, 1.15 mmol) content.

### 2.3 Cathode Processing

To assess the electrochemical stability of the developed polymers, slurries composed of C_65_ and polymers in a weight ratio of 1:1 were prepared and cast on aluminum foil using an automatic film applicator (NEURTEK Instruments, 40 mm s^–1^). After drying at room temperature, disks of 10 mm in diameter were punched, resulting in 1 mg_C_65__ cm^–2^.

The cathode slurries were prepared by dissolving the binder (5 wt %) and by subsequent addition of the active material (90 wt %) and conducting carbon (5 wt %). The mixtures were homogenized using a Speed Mixer (3000 rpm) and cast similarly to C-polymer electrodes. Very similar loadings were achieved by tuning the viscosity and thickness of the coating (see Table 1). Finally, disks of 10 mm diameter were punched and further dried at 60 °C under high vacuum overnight prior to characterization. The electrodes were evaluated in low (∼0.7 mAh cm^–2^) and high (∼1.2 mAh cm^–2^) loadings.

**Table 1:** Slurry Key Parameters for Casting and the Resultant Capacity Loading

	thickness coated (μm)	solid content (%)	mass loading (mg_LFP_ cm^–2^, mAh cm^–2^)
PVDF	125	43	7.0, 1.20
SB	150	49	3.9, 0.66
ISB-10PEG	150	49	7.8, 1.33
ISB-25PEG	150	49	6.5, 1.10

### 2.4 Characterizations

#### 2.4.1 SB Polymer and ISB-xPEG Copolymers: Structural, Thermal, and Mechanical Characterizations

^1^H NMR spectra of the polymers were acquired in DMSO-*d*_6_ (5 mg mL^–1^), using a Bruker Avance 300 MHz spectrometer at 25 °C. The signal of the residual protons of DMSO (δ = 2.50 ppm) was used as the internal reference standard.

Gel permeation chromatography (GPC) was measured in a PL-GPC 50 integrated GPC system (Agilent Technonologies). 0.05 M LiBr (0.05 M) in DMF was used as the mobile phase at a flow rate of 1.0 mL min^–1^. The column was KD-806 M (Shodex) and all of the measurements were performed at 50 °C. The samples were dissolved in the mobile phase at 1 mg mL^–1^. Molecular weights were determined using a calibration curve based on poly­(methyl methacrylate) (PMMA) standards.

Differential scanning calorimetry (DSC) analyses were performed on a TA Instrument DSC 25. The following program was used: heating from 40 to 270 °C, 5 min isotherm, cooling from 270 to −70 °C, 5 min isotherm, and heating from −70 to 270 °C. The heating and cooling rates were 20 °C min^–1^. The thermal stability of the whole set of polymers was investigated by thermogravimetric analysis (TGA) (TGA 8000 Pekin Elmer) under a nitrogen flow (40 mL min^–1^), at a heating rate of 10 °C/min, from 40 to 800 °C.

The rheological properties were determined by using a strain-controlled ARES-G2 rotational rheometer (TA Instruments). Samples of 8 mm diameter were analyzed in a parallel plate geometry. All of the experiments were conducted under linear viscoelastic conditions for the studied temperature range.

#### 2.4.2 SB, ISB-xPEG, and PVDF Binder Performance: Cell Assembly, Electrochemical Characterization, and Morphology of the Cathodes

The lithium metal was cleaned with cyclohexane and a nylon brush inside an Ar glovebox (H_2_O < 0.1 ppm, O_2_ < 0.8 ppm) until a homogeneous and shiny surface was obtained. Disks of 12 mm in diameter were punched for the characterization of the studied polymers. CR2032 coin cells were employed to assemble the different cells using glass fiber (GF) as the separator and 240 μL of LP40. To assess the electrochemical stability of the different polymers, Li||C-polymer cells were used, while Li||LFP cells were employed to evaluate the performance of the polymers as binders for lithium-ion batteries.

The electrochemical stability was evaluated by linear sweep voltammetry at 1 mV s^–1^ from the OCV to 4.5 V vs Li^+^/Li^0^. Galvanostatic charge–discharge was employed to assess the rate capability in the range of 0.1–5*C*. Subsequently, the cells were cycled at 0.1*C* for capacity recovery after the stress suffered at high current densities prior to evaluating the long-term stability at 1*C*. The experiments were carried out at room temperature. The different cathode formulations were launched in duplicated cells.

The surface morphology, internal porosity, and adhesion to the current collector of the electrodes were analyzed using a benchtop Hitachi 3030 scanning electron microscope (SEM). All analyses were performed under low vacuum and at a 15 kV accelerating voltage. All samples were prepared on carbon tape.

## 3 Results and Discussion

### 3.1 SB Polymer and ISB-xPEG Copolymers: Structural, Thermal, and Mechanical Characterizations

To develop a sustainable and competitive binder, we combine soft and hard segments into a poly­(arylene ether sulfone) backbone. The hard segment (composed of isosorbide and aromatic rings from the sulfone moieties) provides structural stability, ensuring electrode integrity during cycling.^27,28^ In contrast, the soft segment (composed of PEG, with a composition of 10 and 25 mol %) enables the polymer matrix to absorb mechanical strain, allowing expansion during cycling. Isosorbide and PEG were added simultaneously in order to obtain random copoly­(arylene ether sulfone) (Figure 1a). The SB (100% ISB, no PEG; Table 2) and ISB-10PEG (90 mol % ISB and 10 mol % PEG; Table 2) membranes are flexible (Figure 1b), while the ISB-25PEG (75 mol % ISB and 25 mol % PEG; Table 2) membrane is both flexible and stretchable, showing a synergistic effect between the soft and hard components of the biobased binder. Furthermore, the enhanced flexibility of the polymer is typically associated with the improved adhesion^29^ (Figure 1c) and cracking resistance, which is fundamental for a binder performance.^30^

**Figure 1:**
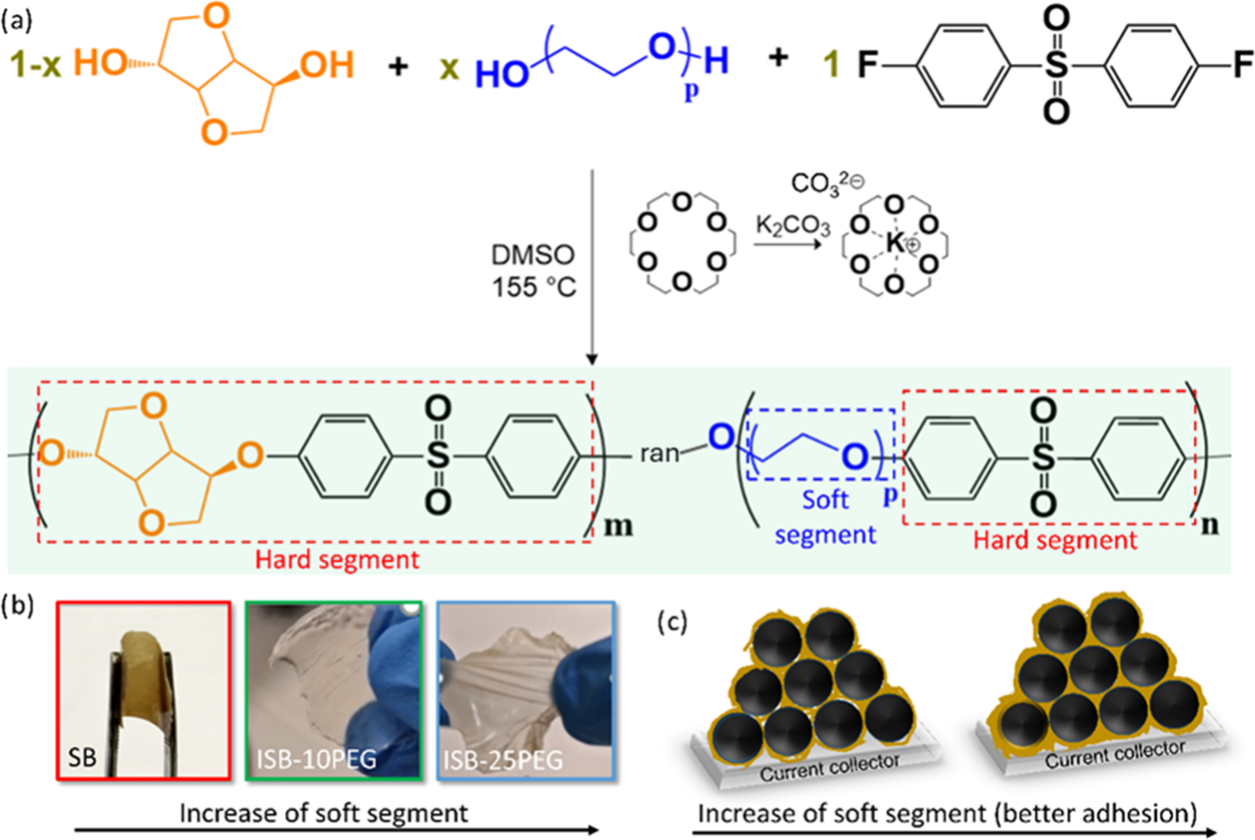
(a) Schematic representation of the synthesis of ISB-xPEG poly­(arylene ether sulfone) copolymers. (b) Photographs of the SB, ISB-10PEG, and ISB-25PEG polymer membranes. (c) Schematic representation of the binder adhesion with an increase of the soft segments in the polymer backbone.

**Table 2:** Nomenclature and Molar and Mass Compositions Determined by ^1^H NMR Spectroscopy^a^

	composition (mol %)^b^		composition (wt %)^c^		GPC			DSC	TGA	
polymer	ISB	PEG	ISB	PEG	*M_n_* (g mol^–1^)	*M*_w_ (g mol^–1^)	*Đ*	*T*_g_ (°C)	*T*_d,5%_ (°C)	residue (%)
SB	100	0	100	0	64,500	125,300	1.94	222	395	24
ISB-10PEG	92	8	74	26	50,200	80,600	1.60	118	372	21
ISB-25PEG	75	25	43	57	17,600	33,400	1.90	0.5	361	26

a Number-average molar mass (*M_n_*), weight-average molar mass (*M*_w_), and molar mass dispersity (*Đ*) were determined by GPC and thermal characterization for ISB, ISB-10PEG, and ISB-25PEG copolymers.

b Calculated by using the eqs S1a-b.

c Calculated by using the eqs S2a-b.

The SB polymer (SB derived from “superbio”)^23^ and the novel ISB-xPEG copolymers were synthesized by aromatic nucleophilic substitution, catalyzed by K_2_CO_3_ and 18-crown-6 (Figure 1a). In this reaction, a complex formed between the K^+^ ion and an alkoxide, with 18-crown-6 playing a key role due to its ability to accommodate the K^+^ ion, resulting in the formation of a “naked” alkoxide ion.^23,31^ This increases the stability of the alkoxide, which is crucial for achieving a high molecular weight and ensuring the success of the reaction. As a result, we obtained a high molecular weight (*M*_w_) (125.3 kg mol^–1^) for the SB polymer, surpassing the entanglement threshold value (28 kg mol^–1^) for isosorbide-based polymers.^32^ Additionally, GPC results (Figure S1 and Table 2) also showed that as the PEG content increases, the *M*_w_ of the copolymer decreases, with values of 80.6 kg mol^–1^ for ISB-10PEG and 33.4 kg mol^–1^ for ISB-25PEG. This decrease in the molecular weight can be attributed to the greater stability of the secondary alkoxides from isosorbide compared to the primary alkoxides derived from PEG.^33^

The chemical structure and composition of the copolymers were confirmed by ^1^H NMR (Figure S2 and Table 2). In all spectra, the presence of signals from aromatic hydrogens (H_h_ and H_g_), isosorbide hydrogens (H_a_, H_b_, H_c_, H_d_, H_e_, and H_f_), and hydrogens from PEG (H_j_) was confirmed. The complete assignment is shown in Figure S2. The main results obtained by ^1^H NMR, GPC, DSC, and TGA are summarized in Table 2.

Thermal properties are also important parameters that are directly imparted in the binder performance and processability. Poly­(arylene ether sulfone) are rigid polymers, which present a high glass transition temperature (*T*_g_) and are used as engineering plastics.^34^ However, to enable processing at milder temperatures, PEG, a flexible polyol, was randomly introduced into the copoly­(arylene ether sulfone) backbone to decrease its *T*_g_. As shown in Figure 2a, a pronounced decrease in the *T*_g_ was observed as the PEG content increases. While SB showed a *T*_g_ of 222 °C, the incorporation of 10 mol % (ISB-10PEG) and 25 mol % (ISB-25PEG) PEG reduces the *T*_g_ to 118 and 0.5 °C, respectively. The stretchability of ISB-25PEG is related to its subambient *T*_g_. As previously mentioned, increasing the PEG content leads to a reduction in the molecular weight (*M*_w_) of the copolymer. While both a lower *M*_w_ and the inclusion of soft segments contribute to the reduction of *T*_g_, the incorporation of PEG has a more pronounced effect. On one hand, the decrease in *T*_g_ due to a lower *M*_w_ is primarily related to increased chain-end mobility and reduced entanglements. On the other hand, the incorporation of soft PEG segments in the polymer backbone presents a plasticizer effect, lowering the activation energy required for conformational changes in the system.^35^ The plasticizing effect caused by the incorporation of soft segments has a more significant impact on the polymer’s final properties than the reduction in the molecular weight (*M*_w_), as illustrated in Figure S3. For instance, the SB polymer with an *M*_w_ of 8000 g mol^–1^ exhibited a *T*_g_ of 68 °C (Figure S3), which is notably higher than that of ISB-25PEG, which has an *M*_w_ of 33,400 g mol^–1^ but a lower *T*_g_ due to the presence of PEG segments. Interestingly, both copolymers showed a single *T*_g_ (indicated by the dotted gray lines), suggesting no phase separation (miscibility between both soft and hard segments). Additionally, the PEG backbone did not crystallize in the copolymers, although both the precursor and the PEG-arylene ether sulfone homopolymer are crystalline solids at room temperature (Figure S4). The same was observed for segmented polyurethanes synthesized using macrodiols with a molecular weight < 2 kDa,^36^ where the random copolymer structure prevented macromolecular packing into crystalline lamellae.

**Figure 2:**
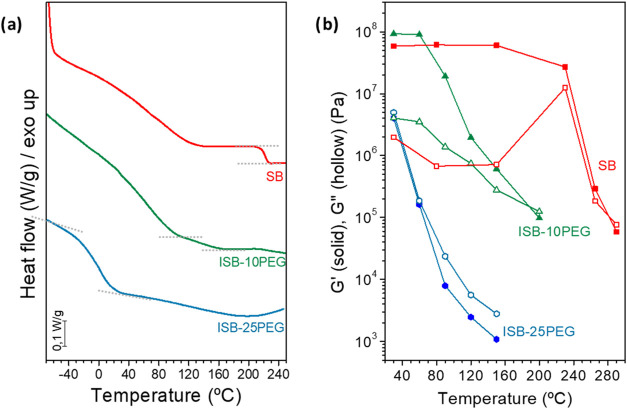
(a) DSC 2nd heating curves and (b) storage (*G*′) and loss (*G*″) moduli as a function of temperature of the SB polymer (red horizontal bar) and the copolymers ISB-10PEG (green horizontal bar) and ISB-25PEG (blue horizontal bar) at 1 Hz and 0.5% strain.

The thermal degradation stability was evaluated, and the results are shown in Table 2 and Figure S5. The SB polymer showed the highest 5 wt % loss temperatures (*T*_d,5%_ = 395 °C). As the PEG content increased, the thermal stability decreased, with the degradation temperatures of ISB-10PEG (*T*_d,5%_ = 372 °C) and ISB-25PEG (*T*_d,5%_ = 361 °C) being lower. However, these degradation temperatures are still higher than those of most biobased commodity polymers (*T*_d,5%_ < 316 °C),^23,37^ though lower than that of PVDF (455 °C).^38^ However, it is important to highlight that these biobased polymers offer more than adequate thermal stability for use as binders in LIBs.

The rheological properties are also influenced by the PEG content, following the same trend as the DSC results shown in Figures 2b and S6. The SB copolymer exhibited a solid-like behavior, with the storage modulus (*G*′) surpassing the loss modulus (*G*″) across the entire frequency range below its *T*_g_. At 230 °C (near its *T*_g_), the loss modulus (*G*″) increased, indicating the transition from a glassy to a viscous state. Above 230 °C, both the storage modulus (*G*′) and loss modulus (*G*″) decreased, approaching similar values, with *G*″ surpassing *G*′ at lower frequencies, signaling a liquid-like behavior (Figure S6a). For ISB-10PEG, at temperatures below 60 °C, both *G*′ and *G*″ were similar to those of the SB polymer. As the temperature increased, both moduli decreased due to the enhanced mobility of the polymer chains. At 200 °C, *G*″ exceeded *G*′, and the material could be processed at lower temperatures than the SB polymer (Figures 2b and S6b). With a further increase in the PEG content (ISB-25PEG), the rheological properties showed a lower temperature for the transition of the elastomer to the thermoplastic response. Both *G*′ and *G*″ decreased gradually with rising temperature due to increased chain mobility, as seen in Figures 2b and S6c.

Based on the polymer characterization, the incorporation of PEG into the polymer backbone lowers the *T*_g_, enhancing the processability of the binder for lithium-ion batteries. Additionally, the PEG segments possess higher polarity and mobility compared to the isosorbide and arylene ether sulfone segments, which can improve both cohesion and adhesion. Consequently, the SB, ISB-10PEG, and ISB-25PEG polymers were evaluated as binders and compared to those of the commercial PVDF binder. The cathode characterization is discussed in the following sections.

### 3.2 Electrochemical Stability

To evaluate the electrochemical stability of the studied polymers for its potential role as biobased binders, active material-free electrodes (polymer-C_65_ 50/50 wt %) were tested as working electrodes versus the Li metal, using a 1 M LiPF_6_ in EC:DEC solution (LP40) as the liquid electrolyte. As shown in Figure 3, the PVDF-based electrode exhibited no degradation peaks across the entire measured voltage range. In contrast, the SB-based electrode displayed a degradation peak at 4.2 V, which shifted to lower voltages with the increasing PEG content (4.13 V for ISB-10PEG and 4.06 V for ISB-25PEG) as expected due to the low electrochemical stability of pure PEG (< 4.0 V).^39^ Consequently, the proposed polymers are suitable for use in sustainable cathode active materials, such as S_8_ and LFP, with operation voltages below 4.0 V.

**Figure 3:**
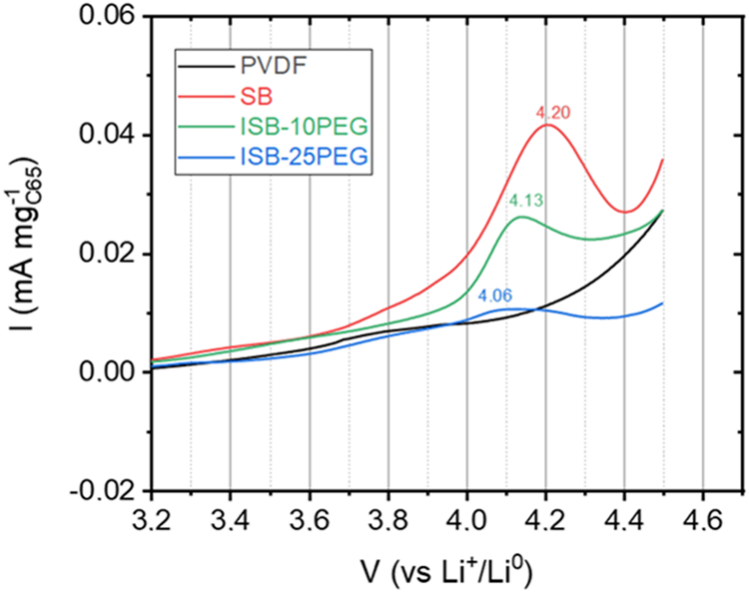
Linear sweep voltammetry curves of Li|GF, 240 μL of LP40|Polymer:C_65_ (1:1 wt %) at 1 mV s^–1^ for PVDF (black horizontal bar), SB (red horizontal bar), ISB-10PEG (green horizontal bar), and ISB-25PEG (blue horizontal bar) polymers.

#### 3.2.1 Application in Li-Ion Batteries

LFP was chosen as the active material to test the performance of the biobased polymers studied as binders in cathodes composed of 90 wt % LFP/5 wt % C_65_/5 wt % polymer with a loading of 1.2 mAh cm^–2^. As shown in Figure S7, SB-based cathodes exhibited significant fissures and began to detach from the current collector at a loading of 0.7 mAh cm^–2^. At a higher loading of 1.2 mAh cm^–2^, the SB binder resulted in poor-quality coatings that detached from the current collector, preventing cell assembly and testing. Consequently, only the cathodes based on ISB-10PEG and ISB-25PEG binders were compared to a reference cathode using a PVDF binder. As shown in Figure 4a–c, SEM images show the particle distribution and integrity of the cathodes. However, the PVDF-based cathodes displayed some cracks, which diminished as the PEG content in the biobased binders increased, with the ISB-25PEG binder demonstrating a homogeneous and robust coating. Additionally, cross-sectional images revealed poor adhesion for the ISB-10PEG and PVDF-based cathodes (Figure 4d,e), while the ISB-25PEG-based cathode exhibited significantly better adhesion and integrity with the current collector (Figure 4f). These results clearly demonstrate that incorporating PEG into the biobased polymer backbone enhances both particle cohesion within the cathodes and adhesion to the current collector. This improvement can be attributed to the increased polarity and mobility of the ISB-xPEG binders, which strengthens intermolecular interactions and adhesion with both the LFP particles and the current collector.^40,41^

**Figure 4:**
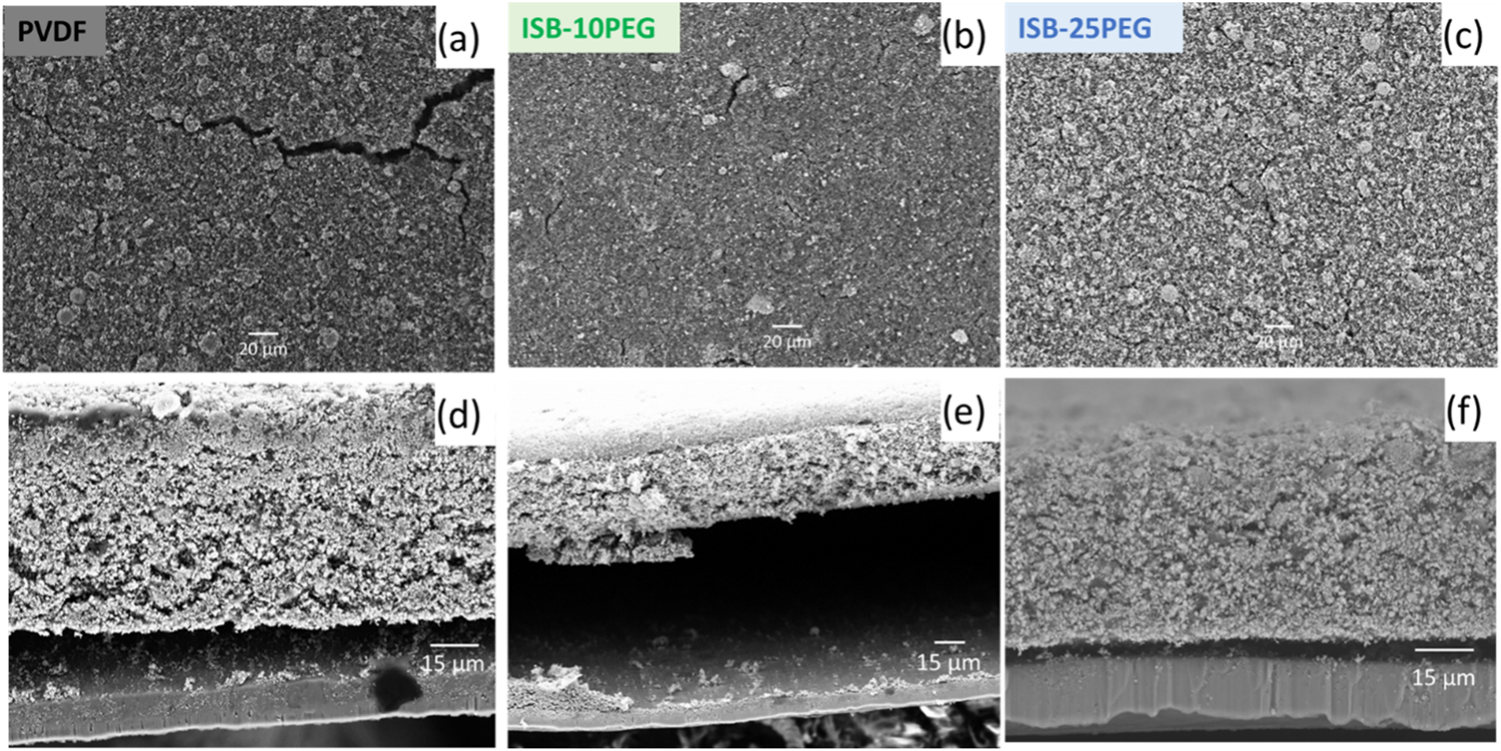
Top-view (upper) and cross-sectional (bottom) SEM images of fresh cathodes (1.2 mAh cm^–2^) based on (a, d) PVDF, (b, e) ISB-10PEG, and (c, f) ISB-25PEG.

The cathodes were cycled in a ramp capability test at different *C*-rates (0.1 to 5*C*), as shown in Figure 5a–c. Notably, the ISB-25PEG cathode achieved the highest Coulombic efficiency in the first cycle (see Figure S8) at 0.1*C* (89.8%), outperforming both the ISB-10PEG (88.6%)- and PVDF-based (87.0%) cathodes. After the first cycle of stabilization, the Coulombic efficiency for all three cathodes remained close to 100%, indicating good reversibility and efficient cycling, reaching very similar capacities at a low *C-*rate (< 1*C*). However, at higher *C*-rates (> 1*C*), a significant capacity drop was observed for all of the cathodes, with the ISB-10PEG-based cathode exhibiting the lowest specific capacity across all *C*-rates. This trend was pronounced at 5*C*, where the ISB-10PEG-based cathode capacity dropped to 37 mAh g^–1^, while the ISB-25PEG- and PVDF-based cathodes maintained capacities of 64 and 69 mAh g^–1^, respectively. To understand the significant drop for the ISB-10PEG-based cathode at 5*C*, electrochemical impedance spectroscopy was used (Figure S9) after rate capability measurements. As a result, the PVDF-based cathode presented the lowest cell resistance (23.3 Ω), followed by ISB-25PEG (31.6 Ω) and finally ISB-10PEG (42.6 Ω). These results match with the capacities observed at high *C*-rates since lower resistances ease a faster lithium-ion insertion. Additionally, the recovery of all cathodes at 0.1*C* after high current density stress suggests that the cathodes were not significantly damaged during high-rate cycling.

**Figure 5:**
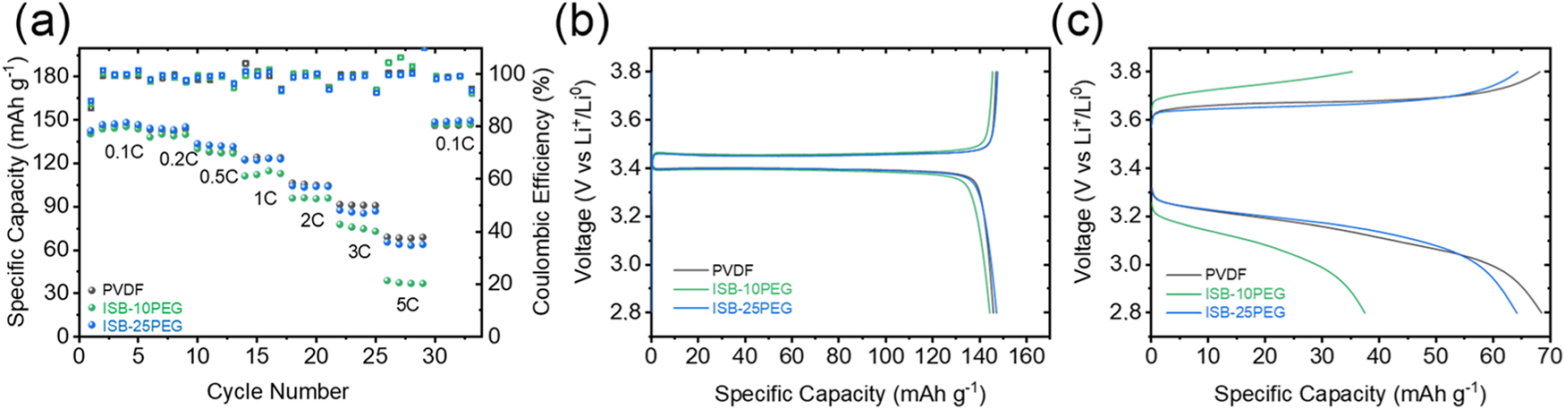
(a) Rate capability of cathodes (1.2 mAh cm^–2^) and voltage profiles of the cells at (b) 0.1*C* and (c) 5*C* rates at room temperature.

After successful *C*-rate capability with no capacity drop at 0.1*C* in the last step, the cells were cycled for long-term stability at 1*C* to assess their cyclability (Figure 6). The results showed that all cathodes retained 80% of their initial capacity at 1*C* after different cycle numbers: the PVDF-based cathode reached this retention after 232 cycles, the ISB-10PEG-based cathode after 241 cycles, and the ISB-25PEG-based cathode after 263 cycles, demonstrating a significant cycle life improvement for the biobased binders. Voltage profiles (Figure 7) further revealed that the PVDF-based cathode experienced a 50% increase in the overpotential between the 50th and 250th cycles, while the ISB-10PEG- and ISB-25PEG-based cathodes showed much smoother increases in the overpotential with rises of 27 and 16%, respectively. These results are consistent with the SEM observations of coating quality and microstructure.

**Figure 6:**
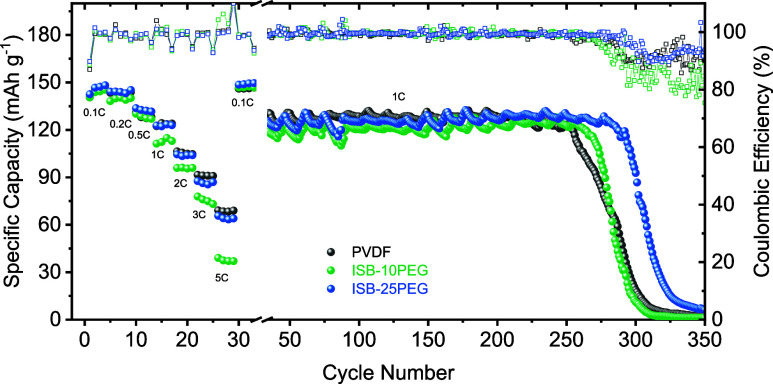
Long-term cycling of cells using different binders: PVDF (black horizontal bar), ISB-10PEG (green horizontal bar), and ISB-25PEG (blue horizontal bar) at the 1*C* rate (1.2 mAh cm^–2^).

**Figure 7:**
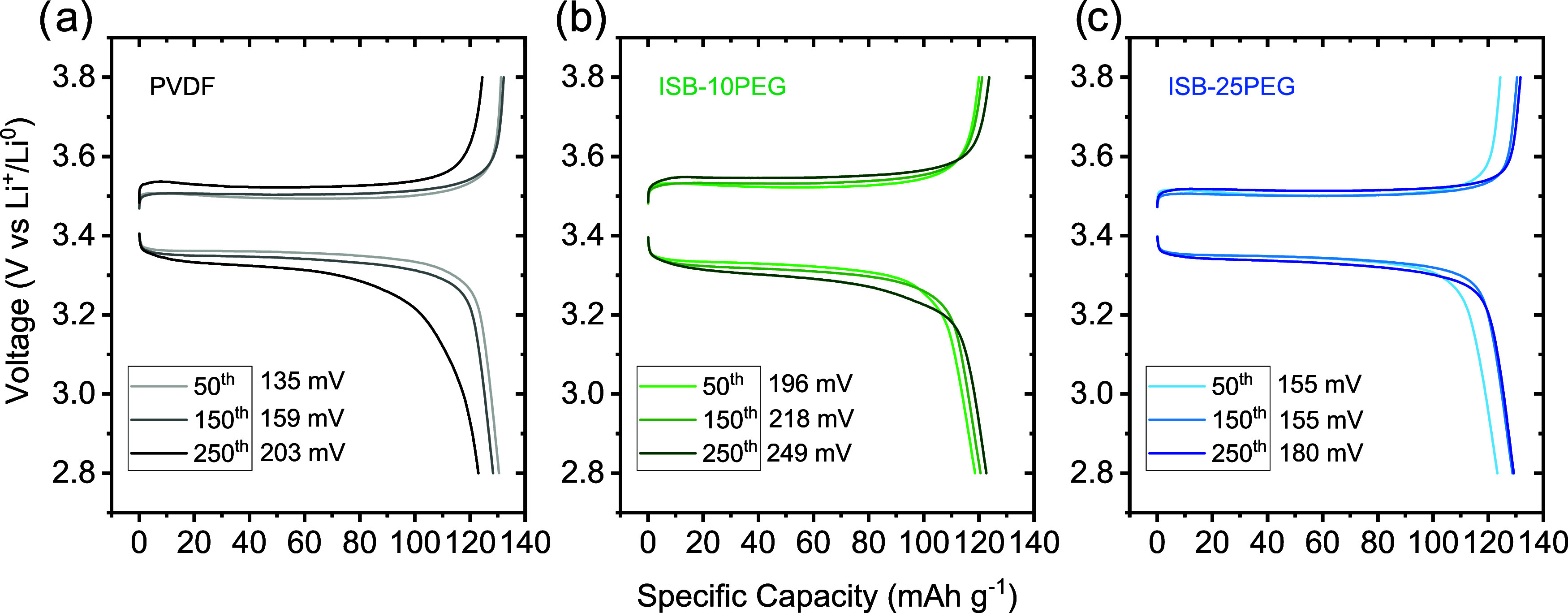
Voltage profiles of the 50th, 150th, and 250th cycles for (a) PVDF-, (b) ISB-10PEG-, and (c) ISB-25PEG-based cathodes at the 1*C* rate.

## 4 Conclusions

Recognizing that the primary challenge in developing a biobased binder lies in obtaining materials with mechanical properties comparable to PVDF—the most widely used binder, which raises environmental concerns due to its fluorinated nature—this work focuses on preparing a poly­(arylene ether sulfone) containing isosorbide and PEG segments. The incorporation of soft (PEG) segments in the hard isosorbide-based poly­(arylene ether sulfone) directly influenced the thermal and mechanical properties of the polymer. All of the polymers were thermoplastic, with the *T*_g_ decreasing from 222 °C (SB polymer) to 118 (ISB-10PEG) and 0.5 °C (ISB-25PEG), thereby enhancing the polymer’s processability. The ISB-25PEG membrane is both flexible and stretchable, showing a synergistic effect between the soft and hard components of the biobased binder.

This synergy was also observed in the LFP cathodes, where the ISB-25PEG-based cathode showed improved mechanical properties compared to the PVDF-based one due to the increased polarity and flexibility imparted by soft PEG segments. This resulted in improved particle cohesion within the cathodes, better adhesion to the current collector, and a more uniform cathode microstructure. At both 0.1 and 5*C* rates, the specific capacities of ISB-25PEG- and PVDF-based cathodes presented were comparable (ISB-25PEG: 146.2 mAh g^–1^ and PVDF: 144.2 mAh g^–1^ at 0.1*C*; ISB-25PEG: 64 mAh g^–1^ and PVDF: 69 mAh g^–1^ at 5*C*). Notably, the ISB-25PEG-based cathode exhibits a superior capacity retention at 1*C*, highlighting a significant improvement in the cycle life, thanks to this biobased binder. These findings contribute to the environmental goals of the battery industry and support the transition toward more sustainable technologies. The promising results lay the foundation for further research and development with the possibility of scaling this technology to broader industrial applications.

## Supplementary Material



## Notes

Some additional data including GPC curves; ^1^H NMR spectra; molar and mass fraction calculations; glass transition temperature data; DSC curves; TGA curves; frequency sweep measurements; SEM images; voltage profiles; and EIS measurements (PDF)
