# Supplementary material for: Innovative Biobased Thermoplastic Binders for Sustainable Lithium-Ion Batteries
Source: ACS Omega. 2025 Jun 2;10(22):22944–51. doi: 10.1021/acsomega.5c00341 (PMC12163790; doi:10.1021/acsomega.5c00341)
Supplement: Supplementary file 1 [file ao5c00341_si_001.pdf]

Innovative bio-based thermoplastic binders for sustainable lithium-ion batteries

Daniela de Morais Zanata,[1] Rafael Del Olmo,[1] Mikel Larumbe,[1] Marcela de Paula Ramos,[1] Nery M. Aguilar,[1] Irune Villaluenga[1,2,*]

[1] POLYMAT, Applied Chemistry Department, Faculty of Chemistry, University of the Basque Country UPV/EHU, 20018 Donostia - San Sebastián, Spain.

[2] IKERBASQUE Basque Foundation for Science 48013 Bilbao, Spain.

*Corresponding Authors: irune.villaluenga@ehu.eus

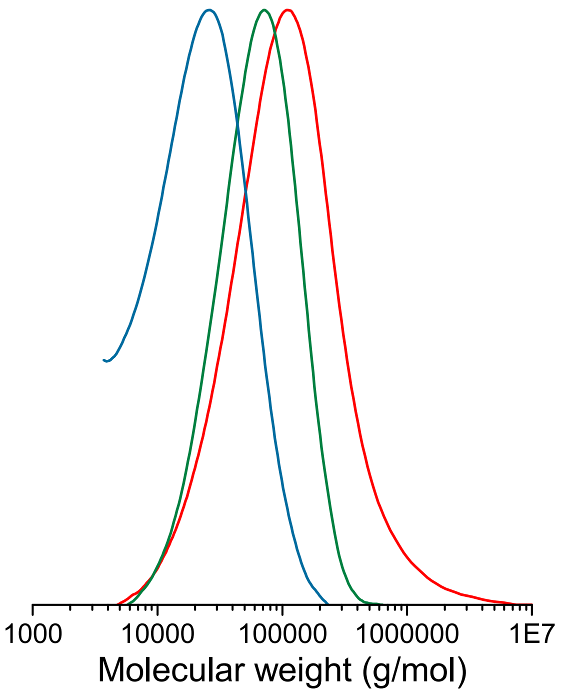

Figure S1. GPC curves of (—) SB homopolymer, and the copolymers (—) ISB-10PEG and (—) ISB-25PEG.

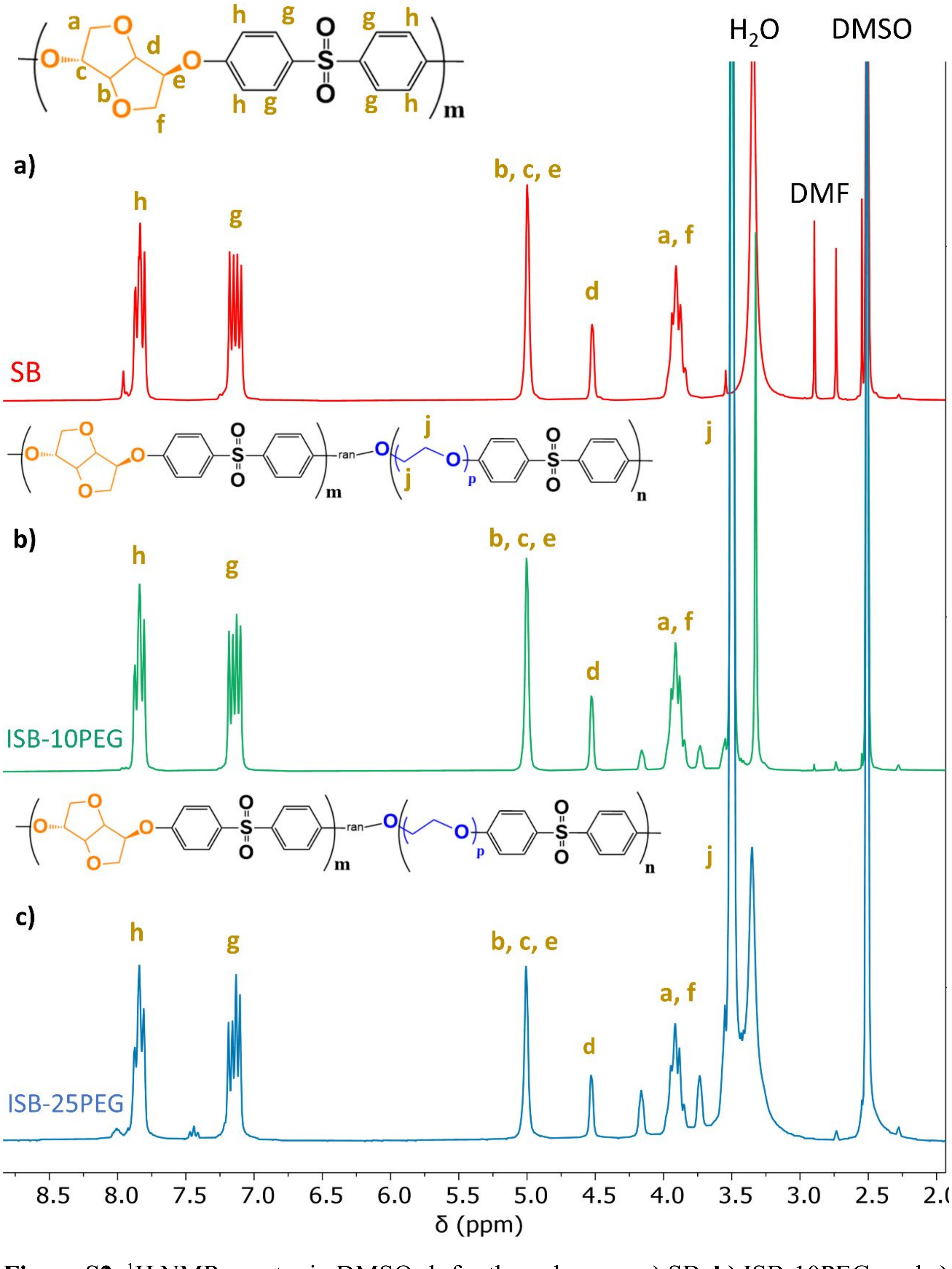

Figure S2. ^{1}H NMR spectra in DMSO-d_6 for the polymers: **a)** SB, **b)** ISB-10PEG, and **c)** ISB-25PEG, as well as their chemical structure with labelled hydrogens.

The molar (f) and mass fraction (ω) of ISB/AES and PEG/AES moieties were calculated by using the Equations S1a-b and S2a-b, respectively.

$$f_{ISB/AES} = \frac{\frac{A_{H_d}}{1}}{(\frac{A_{H_d}}{1})+(\frac{A_{H_j}}{120})}$$ **(Equation S1a)**

$$f_{PEG/AES} = \frac{(\frac{A_{H_j}}{120})}{(\frac{A_{H_d}}{1})+(\frac{A_{H_j}}{120})}$$ **(Equation S1b)**

$$\omega_{ISB/AES} = \frac{(\frac{A_{Hc}}{2} \times M_{OEGMA})}{(\frac{A_{Hc}}{2} \times M_{OEGMA})+(\frac{A_{H_j}}{6} \times M_{DMAEMA})}$$ **(Equation S2a)**

$$\omega_{PEG/AES} = \frac{(\frac{A_{H_j}}{120} \times M_{PEG/AES})}{(\frac{A_{H_d}}{1} \times M_{ISB/AES})+(\frac{A_{H_j}}{120} \times M_{PEG/AES})}$$ **(Equation S2b)**

in which A_{H_d} and A_{H_j} are the areas of the signals assigned to the H_d of the ISB/AES moieties (δ = 4.5 ppm) and the signals assigned to the hydrogens of the ethylene glycol H_j of PEG/AES moieties (δ = 3.5 ppm), respectively. $M_{ISB/AES}$ and $M_{PEG/AES}$ are the molar masses of the ISB/AES (382.39 g/mol) and PEG/AES moieties (1536.25 g/mol), respectively.

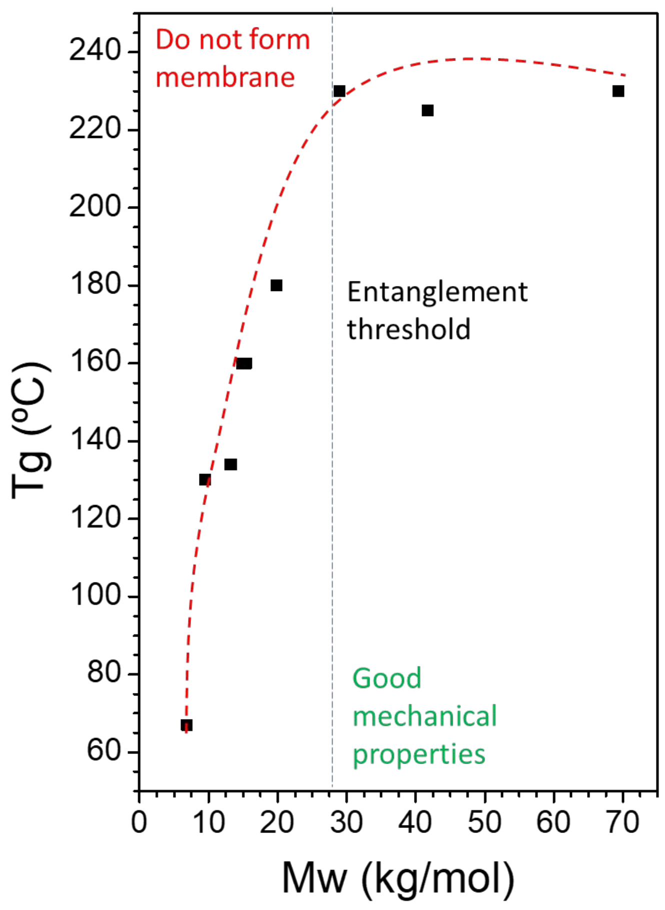

Figure S3. Glass transition temperature (T_g) as a function of the molecular weight for the SB polymer, showing the influence of the entanglement threshold in the mechanical properties.

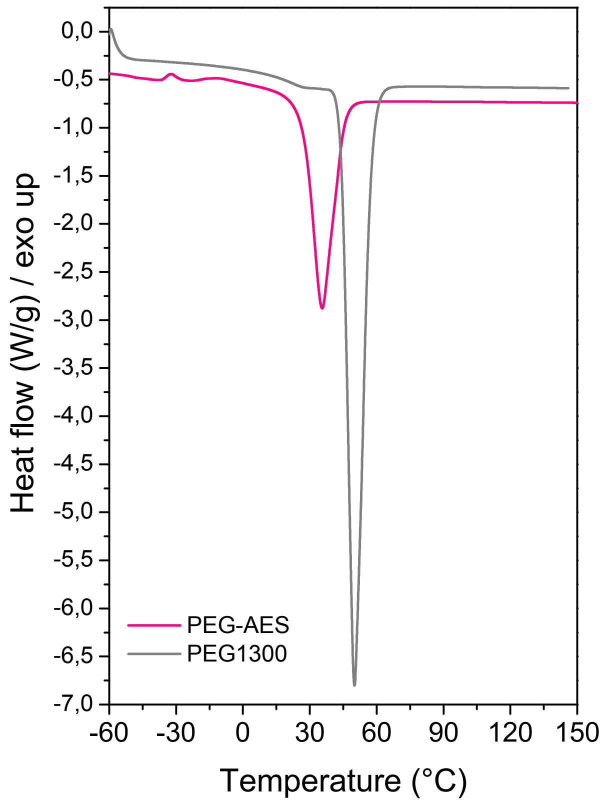

Figure S4. DSC 2nd heating curves of (—) PEG-AES (PEG arylene ether sulfone) and (—) the PEG1300 macrodiol

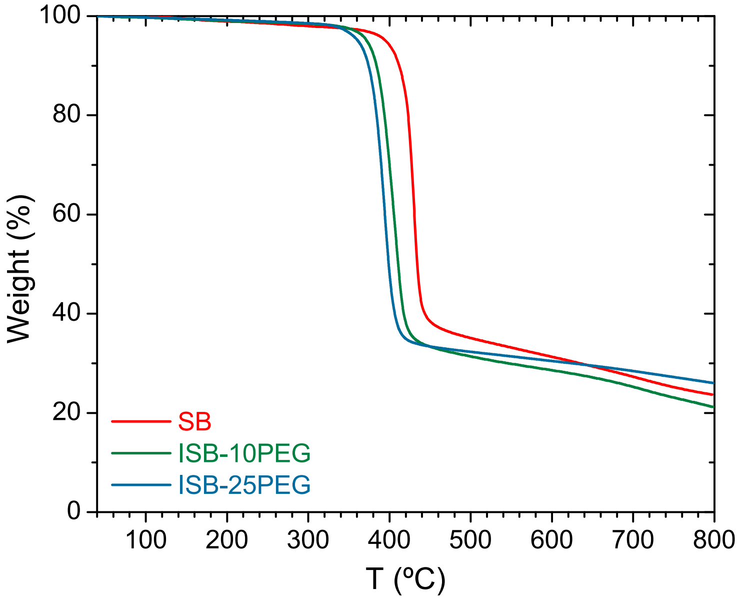

Figure S5. Thermogravimetric curves of (—) SB polymer, and the copolymers (—) ISB-10PEG and (—) ISB-25PEG.

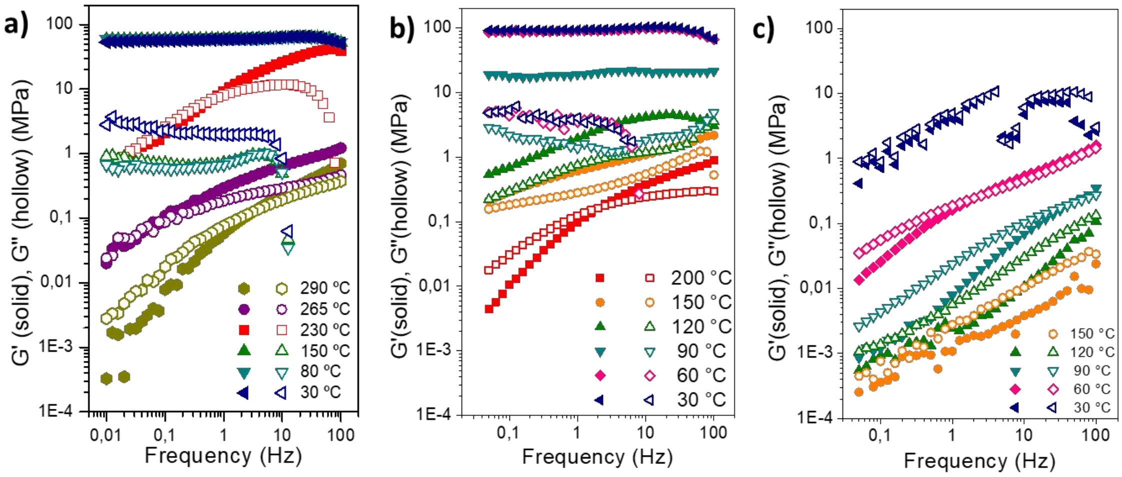

Figure S6. Frequency sweeps measurements for **a)** SB polymer and the copolymers **b)** ISB-10PEG and **c)** ISB-25PEG at 0.5% strain.

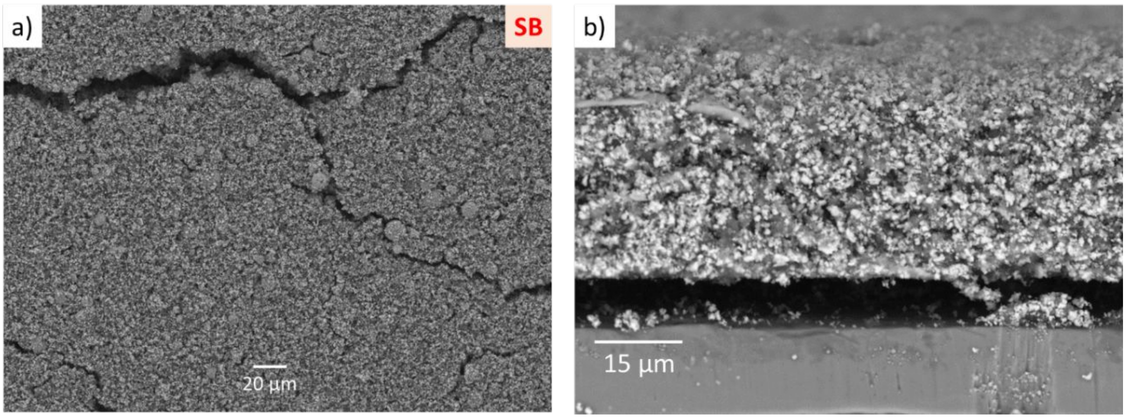

Figure S7. Top-view (a) and cross-sectional (b) SEM images of fresh SB-based electrode (0.7 mAh cm^{-2}).

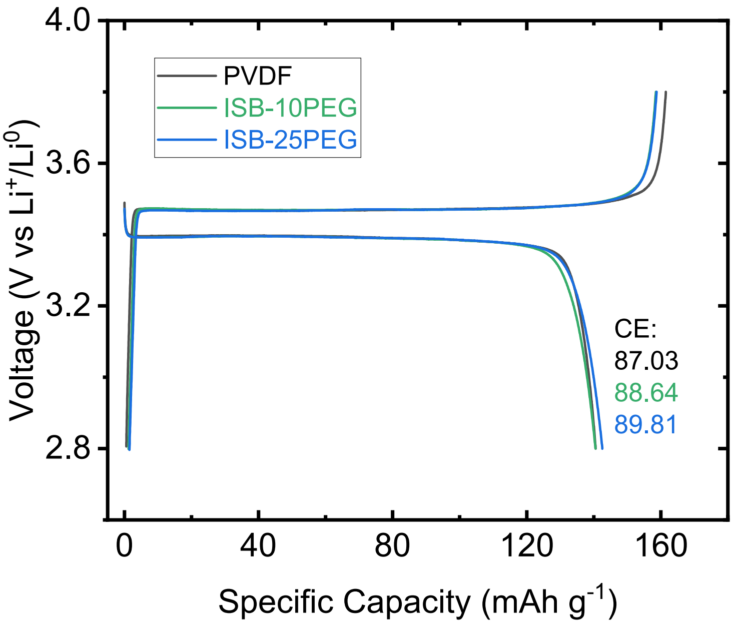

Figure S8. Voltage profile of 1st cycle for PVDF, ISB-10PEG and ISB-25PEG based cathodes in Li||LFP cells at 0.1C and room temperature.

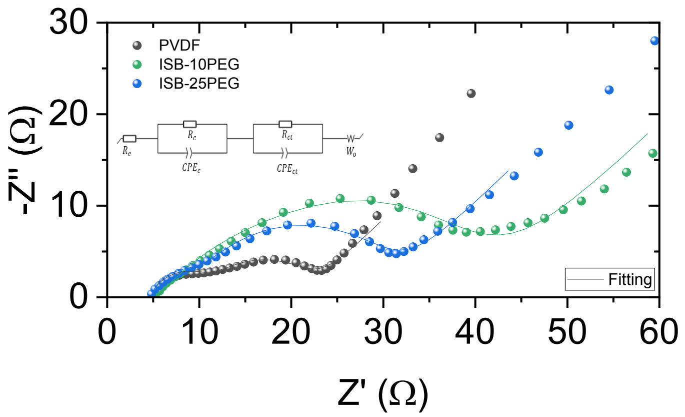

Figure S9. Electrochemical impedance spectroscopy recorded after C rate ramp of the cells containing PVDF, ISB-10PEG and ISB-25PEG binders.
